# Effect of Intermittent Low-Frequency Electrical Stimulation on the Rat Gastrocnemius Muscle

**DOI:** 10.1155/2013/480620

**Published:** 2013-07-09

**Authors:** Arata Tsutaki, Riki Ogasawara, Koji Kobayashi, Kihyuk Lee, Karina Kouzaki, Koichi Nakazato

**Affiliations:** ^1^Graduate School of Health and Sport Science, Nippon Sport Science University, 7-1-1 Fukazawa, Setagaya-ku, Tokyo 158-8508, Japan; ^2^Faculty of Sport and Health Science, Ritsumeikan University, Shiga, Japan

## Abstract

Low-frequency neuromuscular electrical stimulation (NMES) has been used as an endurance exercise model. This study aimed to test whether low-frequency NMES increases the phosphorylation of anabolic signaling molecules and induces skeletal muscle hypertrophy, as seen with high-frequency NMES. Using Sprague-Dawley rats, 1 bout of exercise (with dissection done immediately (Post0) and 3 h (Post3) after exercise) and another 6 sessions of training were performed. All experimental groups consisted of high- and low-frequency stimulation (HFS: 100 Hz; LFS: 10 Hz). Periodic acid-Schiff (PAS) staining was conducted to investigate type II fiber activation, and western blot analysis (WB) was conducted to examine whether NMES leads to anabolic intracellular signaling. At first, we examined the acute effect of exercise. PAS staining revealed that glycogen depletion occurred in both type I and type II fibers. WB results demonstrated that p70S6K phosphorylation was significantly increased by HFS, but there was no significant difference with LFS. In contrast, ERK 1/2 phosphorylation was increased by LFS at Post0. In the 6-session training, the wet weight and myofibrillar protein were significantly increased by both HFS and LFS. In conclusion, LFS has a similar anabolic effect for skeletal muscle hypertrophy as HFS, but the mediating signaling pathway might differ.

## 1. Introduction

Skeletal muscle demonstrates high plasticity, responding to external stimuli with morphological changes. Skeletal muscle hypertrophy is a well-known adaptative response to external stimuli, such as resistance training. For humans, the American College of Sports Medicine recommends resistance training of over 70% of 1 repetition maximum (RM) to lead skeletal muscle hypertrophy [1]. In muscle contraction leading to hypertrophy, type II fibers respond to external stimuli with a greater amount of hypertrophy than do type I fibers [2–4]. Since this process is governed by the size principle, training to induce skeletal muscle hypertrophy demands high force in order to generate type II fibers in voluntary contraction. Conversely, several recent studies have reported that low-intensity (~30% 1 RM) training, in conjunction with high volume, also causes protein synthesis and skeletal muscle hypertrophy [5, 6]. The most convincing hypothesis is that the later phases of exhaustive training regimens also recruit type II fibers, regardless of the training intensity. Taken together, the mobilization of type II fibers can be viewed as being critical for the induction of skeletal muscle hypertrophy.

Muscle hypertrophy is caused by a shift of the protein metabolic balance toward an anabolic state. This mechanism is regulated by intracellular signaling. Recent studies have shown that the mammalian target of the rapamycin complex (mTORC) signaling pathway is a key regulator of the protein synthesis rate and muscle size [7, 8]. Furthermore, the state of phosphorylation of the 70-kDa ribosomal protein S6 kinase (p70S6K), one of the downstream targets of mTORC signaling, is frequently used as an indicator of training-induced hypertrophy [9–11]. The mitogen-activated protein kinase (MAPK) cascade has also been reported to be another anabolic pathway that is activated by mechanical stress, such as muscle contraction modes, and metabolic states [12–14]. Specifically, the activation of extracellular-signal regulated kinase (ERK) 1/2, but not p38 MAPK, is crucial for the induction of muscle hypertrophy [13]. Taken together, the mTORC1 and ERK 1/2 signaling molecules can be regarded as indicators of training-induced muscle hypertrophy.

Electrical stimulation of the skeletal muscle, typically termed neuromuscular electrical stimulation (NMES), is commonly used clinically to enhance the rehabilitation of skeletal muscle function. The force frequency relationship of NMES indicates that increases in the stimulation frequency result in increased muscle force production [15]. In animal experimental muscle models for skeletal muscle hypertrophy, NMES is generally performed with intermittent and high frequencies (more than 60 Hz) [16–19]. On the other hand, when NMES was applied with a lower electrical frequency (~20 Hz) and long activation times, it led to higher oxidative properties and a fast-to-slow conversion of the muscle fiber phenotype [20]. Therefore, animal experimental models with NMES were used to mimic resistance training and/or endurance exercise in order to test the effects of changes in frequency and stimulation time on muscle hypertrophy [21]. Because of its lower exerted force, low-frequency NMES has not been used as a stimulator for muscle hypertrophy. Since NMES randomly activates type I and type II fibers, independent of frequency [22], there might be a possibility that low-frequency NMES will lead to muscle hypertrophy in the same way that high-frequency does. 

In the present study, we aimed to investigate whether intermittent activation with high (100 Hz) or low (10 Hz) frequencies of NMES could induce skeletal muscle hypertrophy in the rat. For this purpose, we conducted 2 experiments. First, we investigated whether acute bouts of exercise induced by low or high electrical frequencies of NMES could sway skeletal muscles toward anabolic states. We conducted histological and western blot analysis, focusing on the intracellular signaling phosphorylations of p70S6K (Thr389) and ERK 1/2 (Thr202/Tyr204). In a second experiment, we subjected rats to 6 sessions of training with low or high electrical frequencies, to investigate whether chronic NMES with different frequencies induces muscle hypertrophy.

## 2. Materials and Methods

### 2.1. Animal Care

Thirty-six male Sprague-Dawley rats (9 weeks old) were purchased from CLEA Japan (Tokyo, Japan). All animals were housed individually, in a 12 h light-dark cycle, with the lights kept on from 6:00 pm to 6:00 am, and they were given food and water ad libitum. All procedures used in this study were approved by the Ethical Committee of the Nippon Sports Science University.

### 2.2. Experimental Design

Rats were divided evenly into a high-frequency electrical stimulation (HFS; 100 Hz) group and a low-frequency electrical stimulation (LFS; 10 Hz) group. They were then further randomly assigned to the following groups: (1) dissection immediately after training (Post0 group), (2) dissection 3 h after training (Post3 group), and (3) dissection after 6 sessions of training (6-session group). Thus, there were 6 groups defined by the frequency of electrical stimulation and the time points at which data were collected (*n* = 6 each group).

### 2.3. Resistance Training Protocol

Under anesthesia, the right lower leg of each rat was shaved. The rats were then subjected to isometric training by electrical stimulation. Each rat was laid prone on a platform and its right knee was extended with a dynamometer, with the ankle joint positioned at an angle of 90°. The triceps of the right leg muscle was then stimulated (voltage: 30–35 V; pulse duration 4 ms: frequency: 100 Hz for HFS group and 10 Hz for LFS group) with a surface electrode (7.5 mm × 7.5 mm) that was connected to an electrical stimulator and isolator (Nihon Koden, Japan). The left medial gastrocnemius muscle served as the untrained (UT) control.

**Figure 1:**
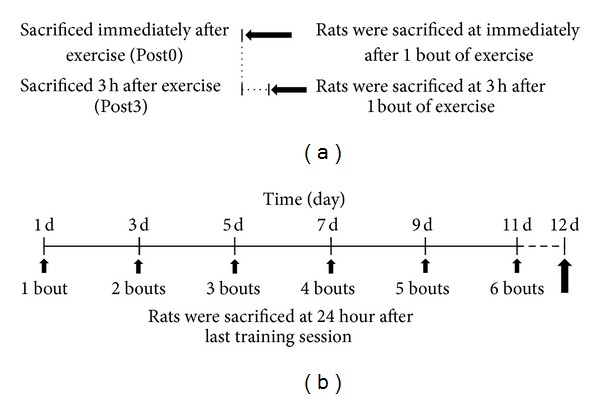
Training protocol. Schematic diagram representing the details of the experimental schedule in this study. Thirty-six Sprague-Dawley rats were used. The rats were divided into 2 groups: high-frequency electrical stimulation (HFS) and low-frequency electrical stimulation (LFS). Rats were subjected to training on every other day. (a) The left illustration represents 1 bout of exercise. (b) The right illustration represents 6 sessions of a training protocol. The rats in all of the experimental groups (*n* = 6) had their medial gastrocnemius muscle extracted after death.

For all training sessions, the triceps surae muscle was trained by stimulation for five 3 s contractions, with a 5 s interval between each contraction. Four sets in total were performed, with 3 min intervals between each set. All training sessions were conducted on every other day. The training and exercise schedule details are presented in Figure 1.

Animals were sacrificed immediately or at 3 or 24 h (6 sessions) after training. The medial gastrocnemius muscle was removed, weighed, and rapidly frozen in liquid nitrogen, and the right and left medial gastrocnemius muscles were triturated in liquid nitrogen and stored at −80°C until use.

### 2.4. Periodic Acid-Schiff (PAS) Staining and Immunohistochemistry

To investigate the intramuscular glycogen, we used a commercially available PAS staining kit (Muto Chemical Co., Ltd., Japan) and performed the protocol according to the manufacturer's instruction manual.

To classify the fiber type, 10 *μ*m thick muscle cryosections were fixed with 2% paraformaldehyde and 0.25% picric acid in 0.1 M phosphate-buffered saline (PBS), for 15 min at room temperature. Following fixation, the sections were washed in 0.1 M PBS for 15 min and then postfixed in ice-cold methanol (−20°C) for 10 min. The sections were then washed 3 times in 0.1 M PBS for 5 min and blocked in a 0.1 M PBS solution containing 5% goat serum and 1% Triton-X-100, for 1 h at room temperature. After blocking, the primary antibodies were applied (laminin and fast myosin heavy chain; Sigma, USA) over night at 4°C. On the following day, the sections were washed in 0.1 M PBS, and the secondary antibody was applied overnight at 4°C. The sections were then washed twice in 0.1 M PBS for 10 min and subsequently dried. Slides were viewed under a light microscope and muscle fibers were classified as either slow myosin heavy chain (MHC I; black) or fast myosin heavy chain (MHC II; green).

### 2.5. Western Blotting Analysis

Frozen muscle powder was homogenized in a buffer containing 50 mM Tris-HCl (pH 7.5), 1 mM ethylenediaminetetraacetic acid (EDTA), 1 mM ethylene glycol tetraacetic acid, 1% Triton X-100, 10% glycerol, protease inhibitor (Roche Applied Science), and phosphatase inhibitor (Thermo Scientific), with sonication for 10 s. Following this, the homogenate was centrifuged for 10 min at 14,000× g, at 4°C, after which the protein extract was mixed with Laemmli sample buffer and then heated to 85°C. Samples were stored at −80°C until use. Equal amounts were loaded onto a gel for electrophoresis, after which the separated proteins were electroblotted onto a nitrocellulose membrane (GE Healthcare) for 90 min.

After blotting, the membrane was washed in Tris-buffered saline containing 25 mM Tris-HCl (pH 7.4), 150 mM NaCl, and 0.1% Tween 20 (TBS-T), blocked with 1% skimmed milk (BD Pharmigen), and then incubated with the desired with the primary antibody overnight at 4°C. On the next day, the membrane was washed with TBS-T, and peroxidase-conjugated secondary antibodies (Thermo Scientific) were applied for 60 min at room temperature. Chemiluminescent reagents (Thermo Scientific) were used for the signal detection. The primary antibodies used in this study were phospho-p70S6K (Thr389), phospho-ERK1/2 (Thr202/Tyr204), and *α*-tubulin (all from Cell Signaling Technology, Japan). Images were then captured, and the signals were quantified using the Ez-capture chemiluminescence detector (ATTO) and CS analyzer software (ATTO). All target proteins were normalized *α*-tubulin expression levels.

### 2.6. Myofibrillar Protein Extraction

Procedures to determine the myofibrillar protein concentration were carried out as described by Karagounis et al. [23]. Approximately 20 mg of powdered muscle was homogenized in a buffer containing 150 mM NaCl, 0.1% Triton-X 100, 20 mM Tris-HCl (pH 6.8), 50 *μ*M dithiothreitol (DTT), 100 mM EDTA, and protease inhibitor (Roche Applied Science) and then centrifuged at 1,600× g for 20 min to produce a myofibrillar pellet. Following removal of the supernatant, the pellet was washed in a low-salt buffer containing 100 mM KCl, 5 mM Tris-HCl (pH 7.4), and 1 mM DTT and centrifuged for 5 min at 13,000× g; the process was repeated twice. The pellet was then washed 2 times in 70% ethanol. After removal of the ethanol, the pellet was resuspended in 0.3 M NaOH, and an aliquot was removed to determine the protein content by using the Lowry assay (RC DC Assay; BioRad). Protein concentrations were corrected for their bovine serum albumin content.

### 2.7. Cross-Sectional Area Measurement

To investigate the cross-sectional area (CSA), we used the immunohistochemical technique described by Bloemberg and Quadrilatero [24]. The primary antibodies used were BA-F8 (MHC I), SC-71 (MHC IIa), BF-35 (MHC IIx), and BF-F3 (MHC IIb), all purchased from the Developmental Studies Hybridoma Bank (University of Iowa). The CSA quantification was performed by a computer application (Winroof; Mitsuya Co., Ltd.), with *n* = 4 in each group. The CSA of each MHC isoform was counted to 100 fibers per sample.

### 2.8. Electrophoresis for Myosin Heavy Chain Composition

The electrophoretic protocol for separation of the myosin heavy chain isoforms was essentially the same as that described by Mizunoya et al. [25]. Muscle powder was homogenized in a buffer of 10% sodium dodecyl sulfate (SDS), 40 mM DTT, 5 mM EDTA, 100 mM Tris-HCl (pH 8.0), and protease inhibitor (Roche Applied Science). The homogenates were centrifuged at 15,490× g for 5 min at 4°C. The supernatant was extracted, and the protein concentration for each sample was determined by ultraviolet absorption spectrophotometry.

Samples were diluted to final protein concentrations of 10–1,280 ng/*μ*L in a mixed sample buffer containing 100 mM DTT, 4.0% SDS, 160 mM Tris-HCl (pH 6.8), 43% glycerol, 0.2% bromophenol blue, and dH_2_O. After boiling, the protein concentrations were adjusted to 20 ng/*μ*L and frozen at −80°C until use.

The separating gel consisted of 30% glycerol and 8% acrylamide, and the stacking gel consisted of 30% glycerol and 4% acrylamide. Electrophoresis was performed at 4°C with a constant voltage of 140 V for 22 h (except for the first 40 min, during which the maximal current was limited to 10 mA to allow stacking gel penetration). The lower running buffer was mixed gently with a magnetic stirrer throughout the entire electrophoresis. After electrophoresis, the gels were stained with a silver staining kit (Silver Stain KANTO III: Kanto Chemicals) and then dried using a Multi gel Dryer (Cosmo Bio Co., Ltd.). Finally, the bands were quantified by densitometry (Cs Analyzer; ATTO).

### 2.9. Statistical Analysis

The statistical differences between the trained (T) and untrained (UT) legs were determined by paired *t*-tests. Concurrently, the statistical differences between HFS and LFS treatments were determined by unpaired *t*-tests. The time course changes in protein expression were examined by a two-way analysis of variance. A post hoc Bonferroni correction was performed using the *t*-test, and all numbers are expressed as the mean ± SD. *P* < 0.05 was considered to denote acceptable significance.

## 3. Results

### 3.1. Experiment I: One Bout of Exercise Induced by NMES with Low and High Electrical Frequencies

#### 3.1.1. Mechanical Parameter of Force Generation

**Figure 2:**
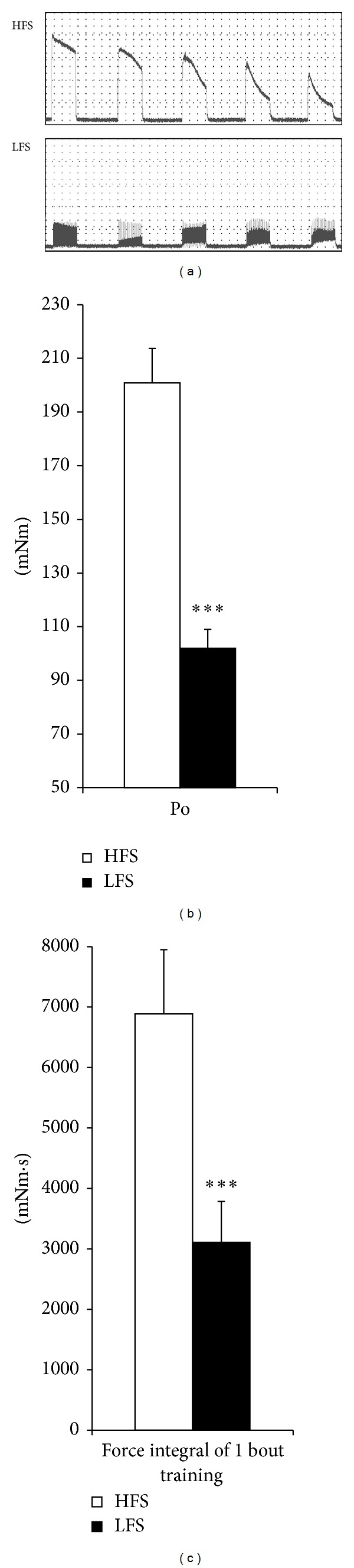
Mechanical parameter of force generation. (a) The typical schema of force generation of each training group. The top figure represents high-frequency electrical stimulation (HFS) and the bottom figure shows low-frequency electrical stimulation (LFS). (b) The peak tetanic torque (Po). (c) The total force generation of 1 bout of exercise. HFS: high-frequency electrical stimulation group. LFS: low-frequency electrical stimulation group. All values are the mean ± SD. ***P < 0.001, versus HFS.

Figure 2 indicates the recorded parameter of force generation from 1 bout of training in the Post0 and Post3 groups.  Figure 2(a) represents the typical schema of force generation in 1 bout of training. The top figure represents the HFS group and the bottom figure represents LFS group.  Figure 2(b) represents the peak torque (Po), and Figure 2(c) indicates the force integral (mNm·S). Both the Po and force integral in the LFS group were significantly lower than the values in the HFS group (*P* < 0.001).

#### 3.1.2. PAS Staining and Immunohistochemistry

**Figure 3:**
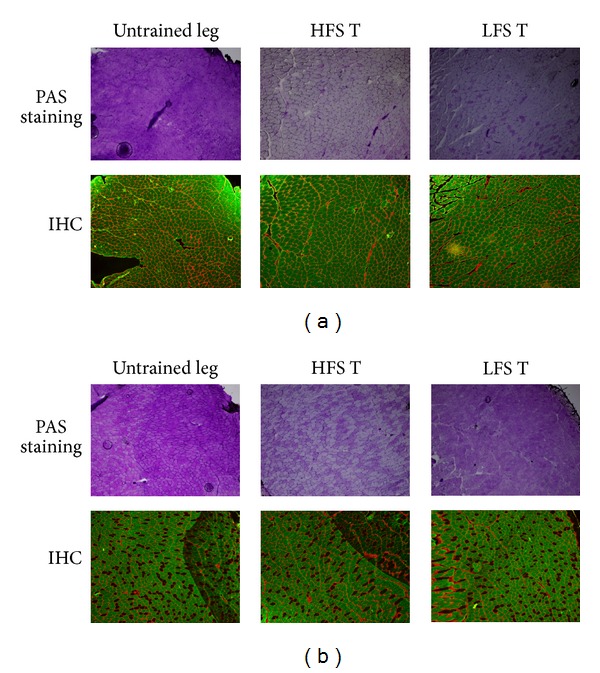
PAS staining and immunohistochemistry. (a) PAS and immunohistochemistry staining patterns in the white portion of the medial gastrocnemius. (b) PAS and immunohistochemistry staining patterns in red portion of the medial gastrocnemius. In PAS staining, the intramuscular glycogen content is indicated by a deep purple color. In immunohistochemistry, myosin heavy chain I (black) and II (green) are indicated. IHC: immunohistochemistry. HFS: high-frequency electrical stimulation group. LFS: low-frequency electrical stimulation group. HFS T and LFS T: the trained leg in HFS and LFS, respectively.

Figure 3 shows the PAS and immunohistochemical staining results for type II fibers of rat skeletal muscle.  Figure 3(a) suggests that type II fibers were dominant in the white portion. On the other hand, Figure 3(b) indicates that both type I and type II fibers coexisted in the red portion.

In Figure 3(a), weak PAS staining was observed in the entire region, suggesting that glycogen depletion occurred under both HFS and LFS. The PAS staining in Figure 3(b) shows weakly stained muscle fibers occurring randomly in type I fibers and type II fibers in both high-frequency- and low-frequency-stimulated muscles, suggesting that electrical stimulation recruited both type I and type II fibers, regardless of the frequency.

#### 3.1.3. Time-Course Changes of Protein Expression

**Figure 4:**
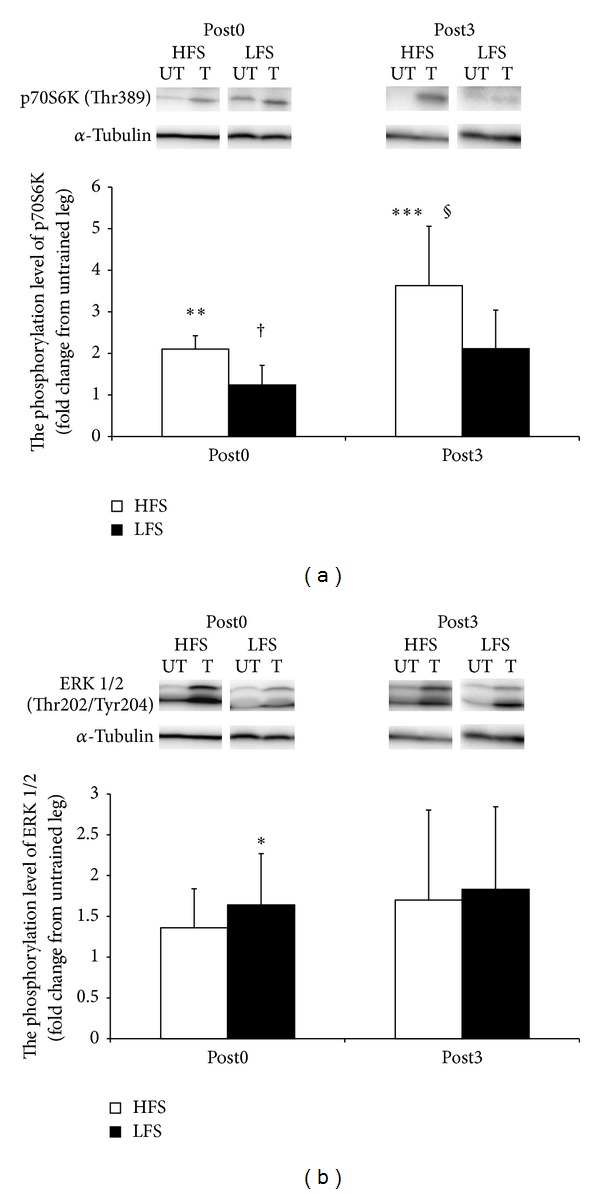
Time-course changes of protein expression. (a) The level of p70S6K phosphorylation at Thr389. (b) The level of ERK 1/2 phosphorylation at Thr202/Thr204. HFS: high-frequency electrical stimulation group. LFS: low-frequency electrical stimulation group. UT: the untrained leg. The relative value was defined as the ratio of trained leg against untrained control leg. All values are the mean ± SD. *P < 0.05, **P < 0.01, and ***P < 0.001 versus UT; ^†^ *P* < 0.05 versus HFS; ^§^ *P* < 0.05, versus Post0.

Figure 4 surveys the change in protein expression over different time points.  Figure 4(a) shows results of the western blot analysis for the state of p70S6K phosphorylation at Thr 389. At both Post0 and Post3, HFS resulted in higher phosphorylation levels than in the UT (*P* < 0.01 at Post0, *P* < 0.001 at Post3; Post0 versus Post3 *P* < 0.05). However, LFS caused no significant variation over either time point. Moreover, the phosphorylation of p70S6K in the LFS group at Post0 was significantly lower than that attributed to HFS at Post0 (*P* < 0.05).

Figure 4(b) represents the state of ERK 1/2 phosphorylation at Thr202/Tyr204. Following HFS, the phosphorylation levels at Post0 and Post3 remained unchanged. However, at Post0, only LFS training produced significantly higher levels of phosphorylation (*P* < 0.05).

### 3.2. Experiment II: Six Sessions of Training with NMES at Differential Frequencies

#### 3.2.1. Physiological Characteristics after Six Sessions of Training Employing Low and High Electrical Frequencies

Table 1 summarizes the results observed after 6 sessions of training with NMES at differential frequencies. No significant change in body weight occurred between the rats treated with HFS and LFS. However, both the HFS and LFS groups exhibited significant increases in both muscle weight (HFS: *P* < 0.01; LFS: *P* < 0.01) and myofibrillar protein content compared with the UT group (HFS: *P* < 0.01; LFS: *P* < 0.05). More importantly, there was no marked difference in these physiological characteristics between the HFS and LFS groups.

#### 3.2.2. Myosin Heavy Chain Composition after Six Sessions of Training

**Figure 5:**
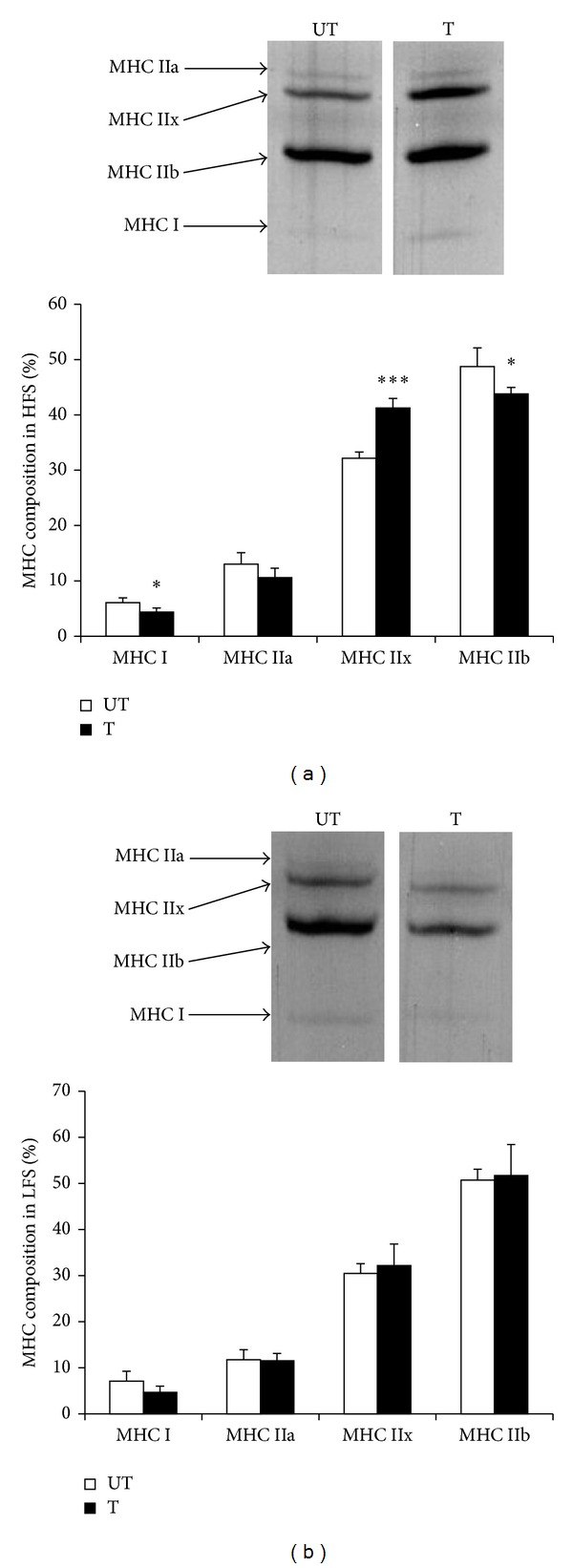
Myosin heavy chain composition after 6 sessions of training. (a) Myosin heavy chain composition after 6 sessions of training in HFS. (b) Myosin heavy chain composition after 6 sessions of training in LFS. HFS: high-frequency electrical stimulation group. LFS: low-frequency electrical stimulation group. T: the trained leg. UT: the untrained leg. MHC: myosin heavy chain. All values are the mean ± SD. *P < 0.05 and ***P < 0.001, versus UT.

Figure 5 indicates the composition of myosin heavy chains after 6 sessions of training with NMES at differential frequencies.  Figure 5(a) shows that HFS significantly decreased the expressions of MHC IIb (*P* < 0.05) and MHC I (*P* < 0.05) and significantly increased the expression of MHC IIx (*P* < 0.01). In contrast, Figure 5(b) reveals that LFS did not change the MHC composition.

#### 3.2.3. Cross-Sectional Area Measurement

**Figure 6:**
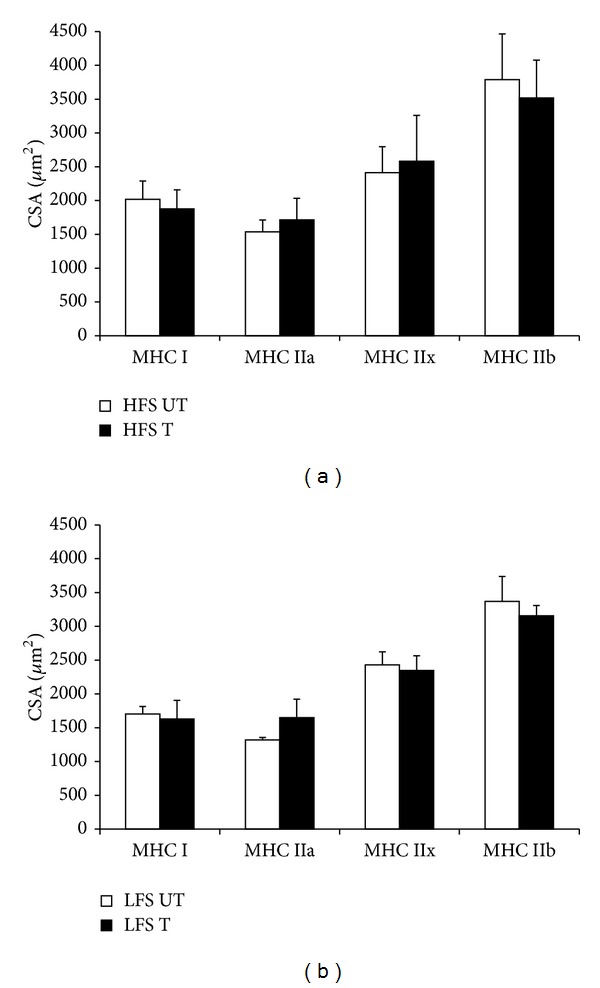
CSA measurement. (a) The CSA after 6 sessions of training in HFS. (b) The CSA after 6 sessions of training in LFS. HFS: high-frequency electrical stimulation group. LFS: low-frequency electrical stimulation group. T: the trained leg. UT: the untrained leg. CSA: cross-sectional area. All values are the mean ± SD.

Figure 6 represents the CSA measurements of the MHC isoforms.  Figure 6(a) represents the HFS group and Figure 6(b) shows the LFS group. No significant change in the CSA of all MHC isoforms was evident in both the HFS and LFS groups.

## 4. Discussion

In this study, we found that 1 bout of intermittent low-frequency electrical stimulations successfully activated one of the anabolic responses of ERK signaling. We also found that chronic bouts of LFS significantly increased the muscle mass and myofibrillar protein. This increase was comparable to that obtained with HFS, which has already been shown as training for muscle hypertrophy. We also found that MHCs and activated signaling molecules differed in composition in LFS- and HFS-induced hypertrophies. In the following discussion, we will discuss the potential biological mechanisms underlying LFS-induced muscle hypertrophy.

A number of studies have demonstrated that the level of p70S6K phosphorylation can be used as an indicator of muscle hypertrophy and/or anabolic response. Our examination found that a significant increase in phosphorylated p70S6K was only observed in the HFS group. Mitchell et al. [6] reported that the level of p70S6K phosphorylation does not increase during low-intensity combined with high-volume training, yet our data showed skeletal muscle hypertrophy occurring with LFS without the phosphorylation of p70S6K. This may be explained by the possibility that our experimental model of LFS represents this low-intensity and high-volume training condition. We also investigated the phosphorylation level of ERK 1/2, a member of the MAPK signaling pathway and found it to be increased only in the LFS group, immediately after training. Since the phosphorylation of p70S6K is elicited mainly in type II fibers [9, 26], the activation of type II fibers and the increase in MHC IIx might be related to signal transduction during HFS. As ERK 1/2 expression did not demonstrate fiber type specificity, our observation that the MHC composition was not affected by LFS stimulation suggests that a relatively high activation of ERK 1/2 might lead to unspecific hypertrophy in rat medial gastrocnemius muscles. Taken together, we speculate that mTOR signaling is a major contributor to HFS-induced hypertrophy, and that MAPK signaling plays a role in LFS-induced hypertrophy.

A key finding in the current study was that the 6-session trainings with LFS successfully induced a significant increase in muscle mass and myofibrillar protein contents, and that this increase was determined to be the same in the muscle stimulated with HFS. Since 1 bout of LFS exercise induces ERK phosphorylation, chronic bout of LFS is an accumulation of such acute anabolic response. However, we failed to observe a significant increase in the CSA analysis after chronic bouts of training. This may be ascribed to the fact that 6-session training is a comparatively shorter time than previous studies [17, 27].

In Section 1, we raised the possibility that NMES randomly activates type I and type II fibers, leading to muscle hypertrophy. In this study, we performed PAS staining to investigate which fibers were activated by NMES. The results showed that muscle fibers were randomly activated by both LFS and HFS, independent of the exerted force. The manner of muscle fiber activation under electrical stimulation was similar to that of a previous report, suggesting that NMES activates muscle fibers at random [15]. Taken together, we consider that both type I and type II fibers are activated under both HFS and LFS treatments.

In both the LFS and HFS groups, the intramuscular glycogen was mainly depleted in the fibers located in the white portion in the medial gastrocnemius muscle. In the red portion, intramuscular glycogen was depleted in the HFS group, but clear staining was observed after LFS treatment. The HFS protocol in our experiment is essentially the same as that used by previous studies [18, 27, 28], and this condition led to a supramaximal activation of gastrocnemius muscles. On the other hand, we observed that the exerted force and work volume in the LFS group was about less than 50% of that in the HFS group. Such differences in exerted force could be related to differences in glycogen consumption.

As seen in the immunostaining of type II fibers, the gastrocnemius muscle consisted mainly of type II fibers, especially in the white portion. This is the same result as shown by previous studies [17, 24, 27]. Since the major fibers were of type II in the white portion, random activation dominantly recruited type II fibers, regardless of the electrical frequency. Since type II fiber activation is a key event in muscle hypertrophy, LFS might successfully induce muscle hypertrophy in gastrocnemius muscles.

Recently, muscle hypertrophy was reported to be induced by moderate- and/or low-intensity (20–50% of 1 RM) resistance training, with blood-flow restriction [29–32]. Moreover, Fujita et al. [33] have also reported that an increase of the muscle protein synthesis rate occurs in the 3 h following low-intensity, blood-flow-restricted resistance exercise. More recent studies have determined that low intensity training to the point of fatigue without blood-flow restriction also elicits a higher protein synthesis rate and muscle hypertrophy [5, 6]. This supports the idea that a recruitment of type II fibers occurs under fatigable conditions, even if the weight lifted is low. It is obvious that human studies are far from the animal models, and our LFS model underscores the theory that activation of type II fibers is a key factor in achieving muscle hypertrophy without high exerted force.

Various exercise methods can induce fiber type alteration. We examined whether training could stimulate fiber type changes, by modifying the myosin heavy chain composition. As previously reported, during HFS, MHC IIx expression was increased in the medial gastrocnemius muscle, whereas MHC IIb and MHC I expressions were decreased [17, 27]. Unexpectedly, we found that LFS did not change the MHC composition. Normally, ATP-consuming muscle contraction induces the switch from a fast- to a slow-twitch fiber activation. Muscle hypertrophy, with no change in the MHC composition, suggests that all muscle fibers are equally enlarged, independent of fiber type. We also speculate that the groups of activated signaling molecules might differ between LFS and HFS. In the acute experiment, we found that p70S6K was activated by HFS, and ERK 1/2 was activated by LFS. Since the phosphorylation of p70S6K elicited mainly type II fibers [9, 26], the activation of type II fibers and the increase in MHC IIx might be related to the signal transduction induced by HFS. As ERK 1/2 expression did not demonstrate fiber-type specificity, our observation that the MHC composition was not affected by LFS suggests that a relatively high activation of ERK 1/2 might lead to unspecific hypertrophy in the rat medial gastrocnemius muscle. It is also important to evaluate whether functional differences exist between muscles treated with different electrical frequencies.

For humans, skeletal muscle activation by NMES is a method of physical therapy aimed at augmenting and/or maintaining skeletal muscle performance. In clinical settings, high-frequency NMES is mostly dedicated to the improvement of the muscular strength and is similar to resistance training [34]. Conversely, low-frequency NMES improves the metabolic and histochemical characteristics of the skeletal muscle and is thought to mimic endurance training [35]. Our data suggest that electrical stimulation of the skeletal muscle with low-force generation can be beneficial in achieving muscle hypertrophy without the pain associated with high frequency electrical stimulation.

## 5. Conclusions

In summary, our present study demonstrates that muscle activation by electrical stimulation recruits type II fibers independently of frequency and that electrical stimulation without high force generation results in muscle hypertrophy. This finding may be applicable to both athletic conditioning as well as to clinical care for sports injuries and muscle atrophy.

## Figures and Tables

**Table 1:** Physiological characteristics after six sessions of training employing low- and high-electrical frequencies.

	HFS	LFS
Body weight (g)	366.36 ± 22.17**	362.31 ± 12.36***
Medial gastrocnemius wet weight (mg)		
T	887.10 ± 20.77	883.92 ± 37.50
UT	832.02 ± 35.54**	823.08 ± 37.48***
Medial gastrocnemius wet weight/Body weight		
T	2.43 ± 0.13	2.44 ± 0.09
UT	2.28 ± 0.11	2.27 ± 0.05
% increase of muscle wet weight (T versus UT)	6.78 ± 5.14**	7.44 ± 3.07*
% increase of myofibrillar protein content (T versus UT)	21.67 ± 13.62	23.80 ± 15.89

HFS: high-frequency electrical stimulation, LFS: low-frequency electrical stimulation, T: trained leg, and UT: untrained leg. Values are mean ± SD. *P < 0.05, **P < 0.01, ***P < 0.001 versus UT.
